# Pink1 protects cortical neurons from thapsigargin-induced oxidative stress and neuronal apoptosis

**DOI:** 10.1042/BSR20140104

**Published:** 2015-02-25

**Authors:** Lin Li, Guo-ku Hu

**Affiliations:** *Department of Bioengineering, College of Materials and Chemistry Chemical Engineering, Chengdu University of Technology, Chengdu, 610059, China.

**Keywords:** apoptosis, phosphatase and tensin homologue (PTEN)-induced kinase 1, neurons, oxidative stress, Cyt-c, cytochrome *c*, ER, endoplasmic reticulum, Pink1, phosphatase and tensin homologue -induced kinase 1, PTEN, phosphatase and tensin homologue, ROS, reactive oxygen species, SOD, superoxide dismutase

## Abstract

Apoptosis mediates the precise and programmed natural death of neurons and is a physiologically important process in neurogenesis during maturation of the central nervous system. However, premature apoptosis and/or an aberration in apoptosis regulation are implicated in the pathogenesis of neurodegeneration. Thus, it is important to identify neuronal pathways/factors controlling apoptosis. Pink1 [phosphatase and tensin homologue (PTEN)-induced kinase 1] is a ubiquitously expressed gene and has been reported to have a physiological role in mitochondrial maintenance, suppressing mitochondrial oxidative stress, fission and autophagy. However, how Pink1 is involved in neuronal survival against oxidative stress remains not well understood. In the present paper, we demonstrate that thapsigargin, a specific irreversible inhibitor of endoplasmic reticulum (ER) calcium-ATPase, could lead to dramatic oxidative stress and neuronal apoptosis by ectopic calcium entry. Importantly, the neuronal toxicity of thapsigargin inhibits antioxidant gene Pink1 expression. Although Pink1 knockdown enhances the neuronal apoptosis by thapsigargin, its overexpression restores it. Our findings have established the neuronal protective role of Pink1 against oxidative stress and afford rationale for developing new strategy to the therapy of neurodegenerative diseases.

## Abstract 2

Pink1 (PTEN-induced kinase 1) have a physiological role in mitochondrial maintenance, suppressing mitochondrial oxidative stress, fission, and autophagy. Our findings indicated that thapsigargin induced oxidative stress and neuronal apoptosis in cultured neurons is at least partly mediated inactivation of Pink1.

## INTRODUCTION

Neuronal death is a major phenomenon in nervous system development and a hallmark of all neurodegenerative diseases. Various modes of neuronal death have been described and are commonly observed in neurodegenerative disorders and stroke [1]. More than half of the initially formed neurons are deleted during normal development through programmed cell death, a phenomenon by which unsuccessfully connected neurons are deleted by apoptosis (cell suicide). The apoptosis is characterized by a sequence of very distinctive morphologic changes in the dying neuron [2,3]. Many of the effectors of this developmental cell-death programme are highly expressed in the developing brain, making it more susceptible to accidental activation of the death machinery. When cells are exposed to various stressors, triggering and potential overshooting of cell-death pathways can occur. In humans, oxidative stress has been implicated in a wide variety of pathologies, including cancer, type II diabetes, arteriosclerosis, chronic inflammatory processes, ischaemia/reperfusion injury and various neurodegenerative diseases [4,5]. Oxidative stress is believed to play important roles in neuronal cell death associated with many different neurodegenerative conditions (e.g., Alzheimer's disease, Parkinson's disease and cerebral ischaemia) and it is believed also that apoptosis is an important mode of cell death in these disorders [6]. Reactive oxygen species (ROS) are particularly active in the brain and neuronal tissue as the excitatory amino acids and neurotransmitters, whose metabolism is factory of ROS, which are unique to the brain and serve as sources of oxidative stress. ROS attack glial cells and neurons, which are post-mitotic cells and therefore they are particularly sensitive to free radicals, leading to neuronal damage. It has been reported that deleterious effects of ROS on human cells may end in oxidative injury leading to programmed cell death, i.e. apoptosis [7,8].

Phosphatase and tensin homologue (PTEN)-induced putative kinase 1 (Pink1) encodes a 581-amino-acid protein with a predicted N-terminal mitochondrial targeting sequence and a conserved serine/threonine kinase domain. Studies have demonstrated that recombinant Pink1 can undergo autophosphorylation as well as phosphorylate generic substrates in vitro [9]. Pink1 mRNA is ubiquitously expressed in human tissues, with highest expression in heart, muscle and testes. It is uniformly expressed in mammalian brain, with highest expression levels found within the cell bodies of neurons and glia [10,11]. Earlier results also showed that Pink1 deficiency results in an age-related loss of neuronal viability and increased sensitivity to stress-induced mitochondrial apoptosis. In keeping with other reports, mitochondrial dysfunction was implicated due to the presence of lowered mitochondrial membrane potential, increased oxidative stress and morphological changes of mitochondria associated with Pink1 deficiency [12,13]. These findings may be of considerable value in understanding of the biologically functions of Pink1.

In the present paper, to further identify the role of Pink1 in neuronal protections against oxidative stress and apoptosis, we investigated the expression alternations of Pink1 under oxidative conditions and whether altered Pink1 expression affected neuron survival. Thapsigargin is a specific irreversible inhibitor of endoplasmic reticulum (ER) calcium-ATPase, which leads ectopic calcium entry and causes oxidative stress and apoptosis [14,15]. We found that thapsigargin could lead to dramatic neuronal apoptosis by promoting oxidative stress in neurons and causes lipid peroxidations and protein oxidative carbonylations. And the neuronal toxicity of thapsigargin inhibits antioxidant gene Pink1 expression. Although Pink1 knockdown enhanced the neuronal apoptosis by thapsigargin, its overexpression restores it. These results were consistent with previous reports, established that Pink1 protects cell from oxidative stress in mammalian cells [11,13,16]. Therefore, Pink1 can be viewed as a potential target for drugs that may be useful to treat pathological conditions associated with diseases of neuronal loss.

## MATERIALS AND METHODS

### Reagents

The inhibitor of ER calcium-ATPase thapsigargin was purchased from Sigma–Aldrich. Neurobasal, B27 and basic fibroblast growth factor were obtained from Life Technologies. Papain was obtained from Worthington and other chemicals from Sigma–Aldrich. The Hoechst kit was from Beyotime Biotechnology Co. The ROS probe for the neuronal ROS detection was from Invitrogen. As for antibodies, the cleaved-caspase 3 antibody was from Cell Signaling Technology. The BcL-2-associated X protein (Bax), B-cell lymphoma 2 (BcL-2), 4-hydroxynonenal (HNE), and 2,4-dinitrophenol (DNP) and Pink1 antibodies were from Abcam. And anti-myc and anti-GAPDH were from Millipore. Other chemicals were of the highest purity available.

### Cortical neuron cultures and pharmacological manipulation

Cultures were prepared from the cortices of embryonic day 18.5 mice and plated in six- or 12-well culture plates or poly-D-lysine coated glass coverslips, according to standard neuronal culture procedures. After 1 day in culture, 10 mM cytosine arabinoside was added for 24 h to prevent glial proliferation. The neurons were subsequently maintained with NeuroBasal medium in 5% CO_2_ at 37°C and used after 9–10 days *in vitro*. For the thapsigargin treatment, the final concentration (1 μM) of thapsigargin was applied to these cells for 30 min and then washed out and re-incubated with neurobasal medium for 4 and 24 h. Equivalent DMSO was used as internal controls. Neurons were plated in six-well plates at 1.0 × 10^5^ cells/ml for Hoechst staining and ROS staining and 1.0 × 10^6^ cells/ml for Western blots and real-time PCR assay. As for the overexpression/knockdown of Pink1 in neurons, it was accomplished by adenovirus transfection. Plasmid vectors expressing wild-type or RNAi Pink1 was constructed using the pDNR-cytomegalovirus (pDNR-CMV) vector (Clontech). The vectors were converted into adenovirus constructs using an Adeno-X Expression System 2 (Clontech) for primary neuron transfections. To confirm the acute effect of Pink1 in neuronal protections, the transfection time is limited to 24 h, under thapsigargin treatment conditions.

### Hoechst staining and ROS staining

For the preparation of Hoechst/ROS staining, neurons were plated with 1.0 × 10^5^ cells/ml in six-well plates. After pharmacological manipulations, cells were directly stained with Hoechst kit from Beyotime for Hoechst staining and ROS probe for ROS staining. The cell counting and fluorescence intensity was quantified through the use of National Institutes of Health software ImageJ.

### Western blots

To extract proteins, cultured neurons were sonicated with lysis buffer (PBS with 1% Triton X-100 and protease inhibitors). The cell lysate supernatants were harvested by centrifugation at 9391 ***g*** for 10 min at 4°C. Protein concentrations of the cell supernatants were evaluated and measured by BCA Protein Assay kit (Thermo Fisher Scientific Inc.). The procedure of Western blots was according to standard protocols. Finally, proteins were detected by Super Signal® ECL (Thermo Scientific Pierce) reagent and exposed to films (Kodak). The protein level quantification was carried out by ImageJ.

### Real-time PCR assay

Total RNAs were extracted from cells using Trizol reagent (Invitrogen), for reverse transcriptions. Quantitative real-time PCR was performed using the Bio-Rad iQ5 system using Bio-Rad proprietary iQ5 software and the relative gene expression was normalized to internal control as GAPDH. Primer sequences for SYBR Green probes of target genes were as follows: superoxide dismutase (Sod)1, CAAGCGGTGAACCAGTTGTG and TGAGGTCCTGCACT GGTAC; Sod2, GCCTGCACTGAAGTTCAATG and ATCTGTAAGCGACCTTGC·C; cytochrome *c* (Cyt-c), TTGTTGGCATCTGTGTAAGAGAATC and GCAAGCATAAGACTGGA CCAAA; Pink1, GCTTTCCCCTACCCTCCAC and GCACTACATTGACCACCG ATT.

### Statistical analysis

All statistical analysis was performed by Image software. Quantitative data were showed in x^−^±s using ANOVA tests for comparisons. The value 0.05 (*), 0.01 (**) and 0.001 (***) was assumed as the level of significance for the statistic tests.

## RESULTS

### Ectopic calcium entry by thapsigargin induces neuronal apoptosis

**Figure 1:**
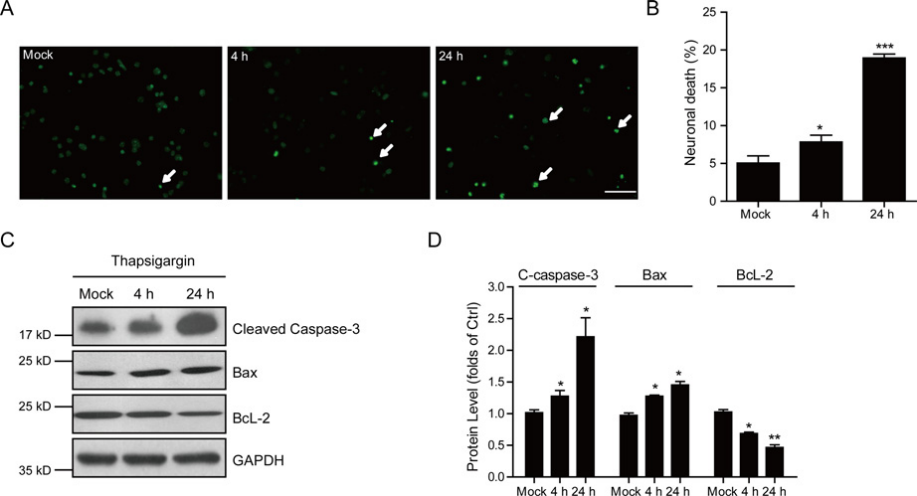
Ectopic calcium entry by thapsigargin induces neuronal apoptosis (**A** and **B**) Hoechst staining and histograms showing the increased cell death (%) after thapsigargin treatment (1 μM for 30 min and recover for 4 or 24 h) in cultured cortical neurons. White arrows indicate apoptotic neurons. Results are averages of three independent experiments. Bar=50 μM. Data represent mean±S.E.M. *P<0.05 and ***P<0.001. (**C** and **D**) Western blots and histograms showing that apoptotic pathways (indicated by increased cleaved caspase-3, Bax and decreased BcL-2) are activated by thapsigargin treatment in cultured cortical neurons. Data represent mean±S.E.M. *P<0.05 and **P<0.01.

Thapsigargin has been reported to induce cell death in several types of cells by increasing either the store-mediated calcium entry or the ER stress [14,17]. To determine whether overwhelmed calcium entry by thapsigargin affects neuronal survival, we quantified neuronal death of cultured cortical neurons under designed growth conditions. After treating cortical neurons with thapsigargin (1 μM) for 30 min and recover for 4 or 24 h, neuronals showed apoptotic morphology detected by Hoechst staining (Figure 1A). By quantifications, we noted that up to 7.84% and 18.9% of neuronals showed apoptotic death at 4 and 24 h by treatment with thapsigargin respectively (Figure 1B). To further confirm the neuronal apoptosis by thapsigargin treatment, we then examined the apoptotic protein alternations. Our results showed the activation of cleaved caspase-3 and Bax, as well as decreased BcL-2 by thapsigargin treatment in neurons (Figures 1C and 1D). Moreover, these results suggested that thapsigargin may arrest neuronal survival in a time-dependent manner in cultured cortical neurons. Taken together, our work establishes that thapsigargin could induce cell apoptosis in cultured neurons.

### Thapsigargin induces dramatic oxidative stress in cultured neurons

**Figure 2:**
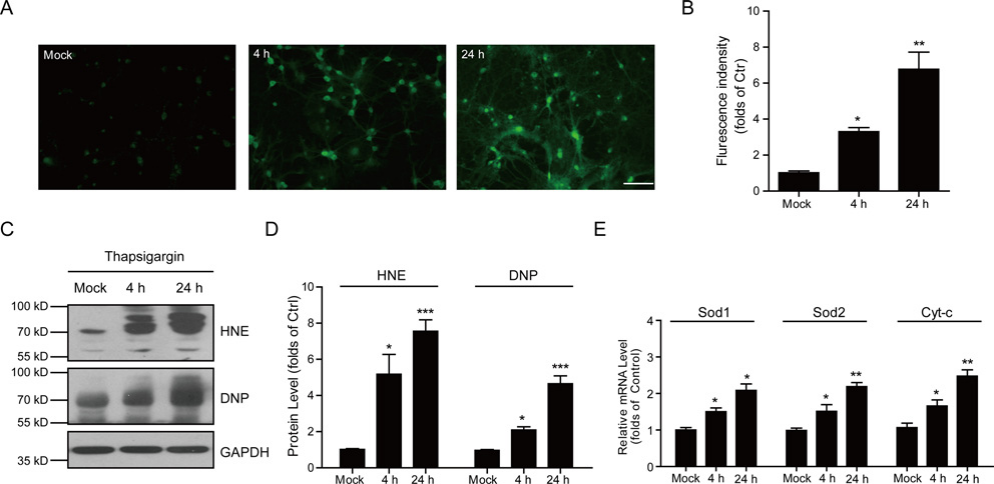
Ectopic calcium entry by thapsigargin increases oxidative stress in neurons (**A** and **B**) ROS probe staining and histograms showing the increased ROS contents after thapsigargin treatment in cultured cortical neurons. Results are averages of three independent experiments. Bar=50 μM. Data represent mean±S.E.M. *P<0.05 and **P<0.01. (**C** and **D**) Western blots and histograms showing the increased HNE and DNP levels by thapsigargin treatment in cultured cortical neurons, indicating oxidative stress. Data represent mean±S.E.M. *P<0.05 and ***P<0.001. (**E**) Histograms showing that the mRNA levels of *Sod1*, *Sod2* and *Cyt-c* are increased by thapsigargin treatment in cultured cortical neurons. Results are averages of three independent experiments. Data represent mean±S.E.M. *P<0.05 and **P<0.01.

To investigate how thapsigargin induces neuronal apoptosis, we focused on the oxidative stress by ectopic calcium entry. Oxidative stress increases ROS production in cells and leads to cell apoptosis. To examine whether thapsigargin induces ROS production in cultured cortical neurons, we applied ROS probe staining to cortical neurons by thapsigargin treatment. The results showed that thapsigargin may significantly induce ROS production for 4 and 24 h (Figure 2A) and the relative fluorescence intensity increased to 3.3-folds (4 h) and 6.8-folds (24 h) (Figure 2B). ROS are generated at the site of inflammation by different mechanisms and one major action is the production of HNE and other reactive carbonyl species, which ultimately promote protein carbonylation [18]. To further confirm the neuronal ROS increasing by thapsigargin, we examined the lipid peroxidation and protein oxidative carbonylation by HNE and DNP antibodies. Our results showed that cortical neurons treatment with thapsigargin (1 μM) induced the expression of HNE and DNP levels (Figures 2C and 2D), indicating that thapsigargin indeed induces oxidative stress. Moreover, we found that thapsigargin treatment also significantly enhanced the mRNA levels of *Sod1*, *Sod2* and *Cyt-c*, which were also oxidative stress markers (Figure 2E). Collectively, all these findings support the notion that thapsigargin may induce ROS production and oxidative stress in a time-dependent manner in cultured neurons.

### Thapsigargin reduces Pink1 neuronal expressions

**Figure 3:**
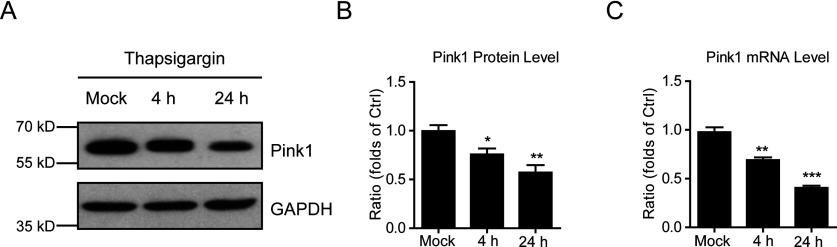
Neuronal Pink1 expression is inhibited by thapsigargin (**A** and **B**) Western blots and histograms showing the protein level of Pink1 is reduced by thapsigargin treatment in cultured cortical neurons. Results are averages of three independent experiments. Data represent mean±S.E.M. *P<0.05 and **P<0.01. (**C**) Histograms showing that the mRNA level of Pink1 is decreased by thapsigargin treatment in cultured cortical neurons. Results are averages of three independent experiments. Data represent mean±S.E.M. **P<0.01 and ***P<0.001.

To study the cellular mechanisms of how thapsigargin affects neuronal survival and induce oxidative stress in cultured neurons, we focused on Pink1. Pink1 contains a putative N-terminal mitochondrial targeting sequence and evidence exists that Pink1 is targeted to mitochondria [9,12]. By Western blots, we found that Pink1 protein levels were reduced by 24.3% and 42.8% after thapsigargin treatment by 1 μM for 4 and 24 h (Figures 3A and 3B). Moreover, the mRNA levels of Pink1 after thapsigargin treatment were also reduced by thapsigargin treatment (Figure 3C) and the changes of mRNA levels were highly correlated with the quantitative changes of protein levels assayed in cultured neurons. These results indicate that thapsigargin may decrease neuronal Pink1 expressions, which may contribute to the neuronal apoptosis.

### Overexpression of Pink1 resists thapsigargin induced neuronal apoptosis

**Figure 4:**
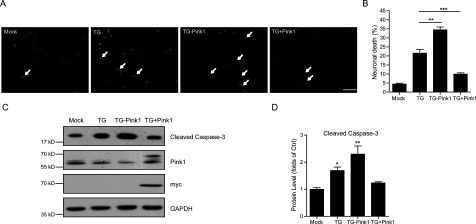
Pink1 neuronal overexpression antagonizes apoptosis caused thapsigargin (**A** and **B**) Hoechst staining and histograms showing the thapsigargin-induced neuronal death is restored by Pink1 overexpression, whereas Pink1 knockdown enhances the neuronal death. White arrows indicate apoptotic neurons. Results are averages of three independent experiments. Bar=50 μM. Data represent mean±S.E.M. **P<0.01 and ***P<0.001. (**C** and **D**) Western blots and histograms showing the thapsigargin-induced cleaved caspase-3 is enhanced by Pink1 knockdown and restored by Pink1 overexpression in cultured cortical neurons, suggesting that Pink1 overexpression could inhibit neuronal apoptosis. Results are averages of three independent experiments. Data represent mean±S.E.M. *P<0.05 and **P<0.01.

To reinforce the possible role of Pink1 in thapsigargin-induced neuronal apoptosis and oxidative stress, we performed to alter Pink1 neuronal expression and examine the correlated apopotsis by thapsigargin. By adenovirus transfections, we found that Pink1 knockdown enhanced the apoptotic effect of thapsigargin on cultured neurons, whereas Pink1 overexpression restored it. Using the Hoechst staining, we noticed that about 21.5% neurons showed apoptotic death by thapsigargin treatment, whereas this value was increased to 34.4% with Pink1 knockdown and reduced to 9.9% with Pink1 overexpression (Figures 4A and 4B). Again, the biochemical results showed that cleaved caspase 3, an apoptotic marker, was induced by Pink1 knockdown and reduced by Pink1 overexpression under thapsigargin conditions (Figures 4C and 4D). Both myc- and Pink1-blots confirmed the Pink1 knockdown/overexpression in these neurons. Taken together, our results showed that Pink1 may be critical for the neuronal protections against apoptosis.

### Overexpression of Pink1 resists thapsigargin-induced oxidative stress

**Figure 5:**
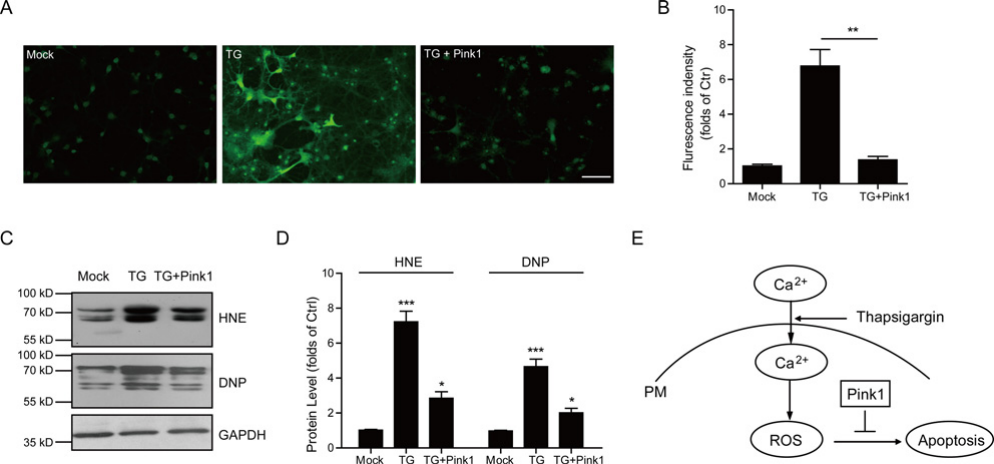
Pink1 overexpression reduces oxidative stress mediated by thapsigargin (**A** and **B**) ROS probe staining and histograms showing the Pink1 overexpression reduce thapsigargin-induced ROS accumulation in cultured cortical neurons. Results are averages of three independent experiments. Bar=50 μM. Data represent mean±S.E.M. **P<0.01. (**C** and **D**) Western blots and histograms showing the HNE and DNP levels are decreased by Pink1 overexpression in cultured cortical neurons. Results are averages of three independent experiments. Data represent mean±S.E.M. *P<0.05 and ***P<0.001. (**E**) Schematic representation highlighting the protective role of Pink1 in ROS-induced neuronal apoptosis. Ectopic calcium entry by thapsigargin induces oxidative stress and leads to neuronal death. Whereas Pink1 overexpression resists ROS accumulation and protects neurons from apoptosis.

We have identified the protective role of Pink1 in neurons against apoptosis and we would like to study whether Pink1 resists neuronal apoptosis via reducing oxidative stress. Thus, we further examined the ROS production in neurons by Pink1 overexpression. The results revealed that the levels of thapsigargin-induced ROS production were significantly reduced by Pink1 overexpression (Figures 5A and 5B). Consistently, oxidative stress indicators HNE and DNP were both significantly decreased by Pink1 overexpression (Figures 5C and 5D). Therefore, all these results strongly indicated that the overexpression of Pink1 may greatly protect neurons from thapsigargin-induced oxidative stress and improve the survival of neuronal cells by inhibit the ROS production.

## DISCUSSION

Apoptosis mediates the precise and programmed natural death of neurons and is a physiologically important process in neurogenesis during maturation of the central nervous system. However, premature apoptosis and/or an aberration in apoptosis regulation are implicated in the pathogenesis of neurodegeneration. In the present study, we demonstrate that thapsigargin could lead to dramatic neuronal apoptosis by ectopic calcium entry. Thapsigargin promotes oxidative stress in neurons and causes lipid peroxidations and protein oxidative carbonylations. Importantly, the neuronal toxicity of thapsigargin inhibits antioxidant gene Pink1 expression. Although Pink1 knockdown enhanced the neuronal apoptosis by thapsigargin, its overexpression restores it (Figure 5E). Our work has established the neuronal protective role of Pink1 against oxidative stress and affords rationale for developing new strategy to the therapy of neurodegenerative diseases.

The relationship between oxidative stress and calcium signals has been extensively studied, given the pathological relevance of the field [19]. An increase in [Ca^2+^] in metabolic situations where the NADH/FADH_2_ production controls the electron transport rate enhances the respiratory flux and ROS production [20,21]. Thapsigargin is a specific inhibitor of the ER calcium ATPase. It causes a transient increase in cytoplasmic Ca^2+^ concentration by inhibiting Ca^2+^ re-uptake. Its high potency and selectivity have been highly valuable in understanding the function of intracellular calcium stores in different processes [22–24]. In the present study, we noticed that thapsigargin may induce ROS accumulation in cultured neurons and the increased HNE and DNP levels were observed.

Under oxidative stress conditions, cells develop a broad range of defence responses to control the cellular ROS level. A first line of defence against ROS includes the superoxide dismutase (SOD) enzymes that catalyse the disproportionation of superoxide to hydrogen peroxide and water [25,26]. In the present study, we also found thapsigargin induced the transcriptions of *sod 1, sod 2* and *cyt-c*, which may be considered as the neuronal intrinsic protective reactions.

Processing of oxygen generates ROS which are involved in intracellular signalling pathways that mediate cellular apoptosis. For example, the Bax gene is an apoptosis-promoting member of the bcl-2 gene family. The Bcl-2 protein is known to form heterodimers with the Bax protein in vivo and the molar ratio of Bcl-2 to Bax determines whether apoptosis is induced or inhibited in several tissues and cells [27,28]. We observed that thapsigargin increases the expression of Bax and reduces the expression of Bcl-2, which may be responsible for the activation of caspase 3 apoptotic cascades.

Pink1 encodes a 581-amino-acid protein with a predicted N-terminal mitochondrial targeting sequence and a conserved serine/threonine kinase domain [16]. Earlier studies indicated that Pink1 plays a physiological role in mitochondrial maintenance, suppressing mitochondrial oxidative stress, fission and autophagy [11,13]. Despite evidences indicating an essential role of Pink1 in cytoprotection, the mechanisms by which Pink1 protects against apoptosis and oxidative stress in neurons are still not well understood. Consistent with the concept that Pink1 is critical for mitochondrial integrity and homoeostasis, we found that the Pink1 may protect neurons against oxidative stress and apoptosis induced by thapsigargin. However, in the current study, we have not determined the role of Pink1 kinase activity in the neuronal protection against thapsigargin. After all, there may be Pink1 functions independent of its kinase activity. We would like to test whether Pink1 kinase activity contributes to its neuronal protective functions in the future work. Nevertheless, our work have identified that Pink1 may deserve to be evaluated as therapeutic agents in oxidant stress-induced neurological disorders.

In summary, our study indicates that thapsigargin induced oxidative stress and neuronal apoptosis in cultured neurons is at least partly mediated inactivation of Pink1. The present findings firstly evaluate the antioxidant and anti-apoptotic effects of Pink1 in cultured neurons, showing that increased Pink1 expression may promote neuron survival. Therefore, Pink1 can be viewed as a potential target for drugs that may be useful to treat pathological conditions associated with diseases of neuronal loss.
